# Consequence of Ischemic Stroke after Coronary Surgery with Cardiopulmonary Bypass According to Stroke Subtypes

**DOI:** 10.21470/1678-9741-2018-0086

**Published:** 2018

**Authors:** Mustafa Aldag, Cemal Kocaaslan, Mehmet Senel Bademci, Zeynep Yildiz, Aydin Kahraman, Ahmet Oztekin, Mehmet Yilmaz, Tamer Kehlibar, Bulend Ketenci, Ebuzer Aydin

**Affiliations:** 1 Department of Cardiovascular Surgery, Istanbul Medeniyet University Medical Faculty, Istanbul, Turkey.; 2 Department of Neurology, Siyami Ersek Thoracic and Cardiovascular Surgery Training and Research Hospital, Istanbul, Turkey.; 3 Department of Cardiovascular Surgery, Siyami Ersek Thoracic and Cardiovascular Surgery Training and Research Hospital, Istanbul, Turkey.

**Keywords:** Stroke, Coronary Artery Bypass, Coronary Artery Bypass/Adverse Effects, Cardiopulmonary Bypass

## Abstract

**Introduction:**

The aim of this study was to determine the outcomes of patients developing ischemic stroke after coronary artery bypass grafting (CABG).

**Methods:**

From March 2012 to January 2017, 5380 consecutive patients undergoing elective coronary surgery were analyzed. Ninety-five patients who developed ischemic strokes after on-pump coronary surgery were included in the study, retrospectively. The cohort was divided into four subgroups [total anterior circulation infarction (TACI), partial anterior circulation infarction (PACI), posterior circulation infarction (POCI), and lacunar infarction (LACI)] according to the Oxfordshire Community Stroke Project (OCSP) classification. The primary endpoints were in-hospital mortality, total mortality, and survival analysis over an average of 30 months of follow-up. The secondary endpoints were the extent of disability and dependency according to modified Rankin Scale (mRS).

**Results:**

The incidence of stroke was 1.76% (n=95). The median age was 62.03±10.06 years and 68 (71.6%) patients were male. The groups were as follows: TACI (n=17, 17.9%), PACI (n=47, 49.5%), POCI (n=20, 21.1%), and LACI (n=11, 11.6%). Twenty-eight (29.5%) patients died in hospital and 34 (35.8%) deaths occurred. The overall mortality rate of the TACI group was significantly higher than that of the LACI group (64.7% *vs.* 27.3%, *P*=0.041). The mean mRS score of the TACI group was significantly higher than that of the other groups (*P*=0.003).

**Conclusion:**

Patients in the TACI group had higher in-hospital and cumulative mortality rates and higher mRS scores. We believe that use of the OCSP classification and the mRS may render it possible to predict the outcomes of stroke after coronary surgery.

**Table:** 

Abbreviations, acronyms & symbols	
CABG	= Coronary artery bypass grafting
CPB	= Cardiopulmonary bypass
ICU	= Intensive care unit
LACI	= Lacunar infarction
LVEF	= Left ventricular ejection fraction
mRS	= Modified Rankin Scale
OCSP	= Oxfordshire Community Stroke Project
PACI	= Partial anterior circulation infarction
POCI	= Posterior circulation infarction
TACI	= Total anterior circulation infarction

## INTRODUCTION

Stroke is a devastating complication of coronary bypass surgery, significantly increasing mortality, morbidity, cost, and the need for long-term care, and reducing the quality of life. The incidence of stroke after cardiac surgery has ranged from 0.4-14% in different series^[1,2]^ and varied by the type of surgery, with concomitant and valvular procedures being associated with the greatest risks^[3]^. However, the incidence of stroke after coronary surgery has been reported to be as low as 1-5% in some previous studies^[4,5]^.

Three different mechanisms may be in play when postoperative stroke develops after cardiac surgical intervention: a brain perfusion disorder, an embolic event, and/or an inflammatory response^[6]^. Early stroke may be caused by an embolism triggered by manipulation of the heart and aorta, or by particles transported via cardiopulmonary bypass (CPB); later events may be associated with low cardiac output, myocardial infarction, atrial fibrillation, and/or hypercoagulability^[7]^. Patients at an increased risk for stroke after cardiac surgery include those with carotid stenosis, peripheral vascular disease, hypertension, diabetes mellitus, renal failure, and/or a recent myocardial infarction^[8]^.

Stroke is a highly heterogeneous disorder with several distinct subtypes, each with specific clinical features. Subtype characterization of stroke may improve our knowledge of outcomes. Several classification systems for ischemic stroke exist, but the most commonly used is the Oxfordshire Community Stroke Project (OCSP) classification (also known as the Bamford or Oxfordshire classification), which clinically recognizes four subtypes of stroke: total anterior circulation infarction (TACI), partial anterior circulation infarction (PACI), posterior circulation infarction (POCI), and lacunar infarction (LACI)^[9]^.

Although previous studies focused on risk factors for stroke after coronary surgery^[10,11]^, only limited data are available on long-term postoperative survival and it is difficult to predict the clinical outcomes of patients who suffer strokes after coronary artery bypass grafting (CABG). We hypothesized that the subtypes of ischemic stroke developing after coronary surgery might affect both mortality and morbidity. Using a simple classification of stroke that is based on clinical findings may aid surgeons to predict the risks of mortality and morbidity, and other patient outcomes.

The aim of this study was to explore the outcomes of ischemic stroke after coronary surgery by OCSP subtype; we measured in-hospital and long-term survival, and stroke disability and dependence scores employing the modified Rankin Scale (mRS), which is widely used worldwide to assess stroke outcomes^[12]^.

## METHODS

### Study Sample

From March 2012 to January 2017, in a single center, 5380 consecutive patients undergoing coronary surgery were retrospectively analyzed. Ninety-five patients who developed ischemic stroke after elective and on-pump CABG surgery were included in the study. The cohort was divided into four subgroups (TACI, PACI, POCI, and LACI) by the subtype of ischemic stroke using the OCSP classification. Exclusion criteria were: 1- patients who had carotid stenosis more than 50 percent, 2- presence of prior stroke, 3- off-pump surgery, 4- re-operation surgery, 5- additional procedure to CABG.

### Data Collection

Preoperative, operative, and postoperative variables including age, gender, left ventricular ejection fraction (LVEF) (%), any history of previous myocardial infarction, diabetes mellitus, hypertension, chronic obstructive pulmonary disease, peripheral vascular disease, and chronic renal failure status, the presence of preoperative atrial fibrillation, cross clamp and CPB time, time of stroke occurrence, lengths of intensive care unit (ICU) and hospital stays, and mortality, were recorded. Data were collected from hospital database and patient's medical records. All postoperative strokes were diagnosed by the same staff neurologist with the aid of brain computed tomography. The interval from stroke to death or follow-up was calculated for each patient.

### Outcomes

The primary endpoints of the study were in-hospital mortality, overall mortality, and later survival in terms of OCSP classification; the average follow-up time was 30 months. The secondary endpoints were the extent of disability and dependency, scored using the mRS.

### Definitions

Stroke was defined as a definite new stroke after cardiac surgery if evidence of sudden onset's neurological symptoms (aphasia, dysarthria, diplopia or hemiparesis) lasted> 24h and confirmed by a brain computed tomography scan. Early stroke was defined as symptoms observed within 48 hours in the course of ICU (n=56), whereas delayed stroke was defined as a detected stroke after 48 hours postoperatively until discharged (n=39).

mRS is a commonly used global scale for measuring the degree of disability and dependency of the patients who have suffered from stroke. The scale runs from 0 to 6: 0= No symptoms; 1= No significant disability. Able to carry out all usual activities; 2= Slight disability. Able to look after own affairs without assistance; 3= Moderate disability. Requires some help, but able to walk unassisted; 4= Moderately severe disability. Unable to attend to own bodily needs without assistance, and unable to walk unassisted; 5= Severe disability. Requires constant nursing care and attention, bedridden, incontinent; 6= Dead.

Mortality: Any reason of dead after coronary bypass surgery in cohort.

### Statistical Method

Statistical analyses were performed using the statistical software NCSS (Number Cruncher Statistical System) version 2007 (Kaysville, Utah, USA). Descriptive data for continuous variables were presented as mean and standard deviation, numbers (%) or median and interquartile range. Differences between the groups were investigated using Student's t-test or the Mann-Whitney test, and Pearson x^2^ test. The survival rates were estimated according to the Kaplan-Meier method. Value of *P*<0.05 was considered statistically significant.

### Ethics Statement

The study was approved by the hospital local scientific ethics committee (Dr. Siyami Ersek Thoracic and Cardiovascular Surgery Hospital, YON.FR.16, 05.12.2016).

## RESULTS 

During the study period, 5380 consecutive patients who underwent elective CABG surgery were analyzed, of whom 95 developed postoperative strokes; all were retrospectively included.

The median cohort age (n=95) was 62.03±10.06 years (range 28-87 years) and 68 (71.6%) patients were male. The clinical OCSP classifications were as follows: TACI (n=17, 17.9%), PACI (n=47, 49.5%), POCI (n=20, 21.1%), and LACI (n=11, 11.6%). The descriptive characteristics of the study sample were shown in Table 1. The overall stroke incidence was 1.76% (95 of 5380).

**Table 1:** Descriptive characteristics of the cohort (n=95).

Variables		Min-Max	Mean±SD
Age (years)		28-87	62.03±10
ICU stay time (days)		0-46	10±12
Postoperative follow (months)		Jan-43	30.1±1.6
Modified Rankin Scale Score		0-6	3.4±2
		**n**	**%**
Gender	Male	68	71.6
	Female	27	28.4
Stroke timing	<48 hours	56	58.9
	>48 hours	39	41.1
In-hospital mortality	Yes	28	29.5
Overall mortality	Yes	34	35.8
OCSP classification	TACI	17	17.9
	PACI	47	49.5
	POCI	20	21.1
	LACI	11	11.6

ICU=intensive care unit; LACI=lacunar infarction; OCSP=Oxfordshire Community Stroke Project classification; PACI=partial anterior circulation infarction; POCI=posterior circulation infarction; TACI=total anterior circulation infarction

As shown in Table 2, which demonstrate the patients characteristics and preoperative data according to OCSP subgroups, concomitant diseases were hypertension (n=85, 89.4%), diabetes mellitus (n=48, 50.5%), peripheral vascular disease (n=48, 32.6%), chronic obstructive pulmonary disease (n=26, 27.3%), and atrial fibrillation (n=12, 12.6%). The mean preoperative hematocrit was 39±4%, the mean creatinine level 1.1±0.7 mg/dL, and the LVEF 52±5.9%. There were no significant differences in patients' preoperative parameters including, preoperative LVEF (*P*=0.392), hematocrit (*P*=0.274), or creatinine level (*P*=0.375), between the OCSP stroke subtypes.

**Table 2:** Patients characteristics and preoperative data according to OCSP groups.

		OCSP Classification				
TACI (n=17)	PACI (n=47)	POCI (n=20)	LACI (n=11)	P*
Age (years)	Mean±SD	58.8±10.9	62.8±9.3	61.5±12.2	64.2±6.8	0.450
	Min-Max (Median)	28-76 (60)	44-80 (64)	34-87 (65)	57-75 (63)	
Gender	Male	11 (64.7%)	34 (72.3%)	14 (70%)	9 (81.8%)	0.793
	Female	6 (35.3%)	13 (27.7%)	6 (30%)	2 (18.2%)	
LVEF (%)	Mean±SD	54.4±5.8	51.1±7.4	53.2±8.3	48.8±8.1	0.392
Hematocrit (%)	Mean±SD	38.1±4.5	39.5±4.1	37.2±5.1	38.7±4.8	0.274
Creatinine (mg/dl)	Mean±SD	0.9±0.3	1.1±0.5	1.3±1.1	1.1±0.3	0.375
Atrial fibrillation	Yes	2 (11.8%)	6 (12.8%)	3 (15%)	1 (9.1%)	0.394
Diabetes mellitus	Yes	7 (41.2%)	26 (55.3%)	8 (40%)	7 (63.6%)	0.445
Hypertension	Yes	16 (94.1%)	42 (89.4%)	17 (85%)	10 (90.9%)	0.366
Previous MI	Yes	9 (52.9%)	32 (68.1%)	10 (50%)	10 (90.9%)	0.092
Peripheral vascular disease	Yes	7 (41.2%)	17 (36.2%)	6 (30%)	4 (36.3)	0.916
Chronic pulmonary disease	Yes	7 (41.2%)	15 (31.9%)	3 (15%)	1 (9.1%)	0.112

LACI=lacunar infarction; LVEF=left ventricular ejection fraction; MI=myocardial infarction; OCSP=Oxfordshire Community Stroke Project classification; PACI=partial anterior circulation infarction; POCI=posterior circulation infarction; TACI=total anterior circulation infarction

* Pearson chi-squared test used.

In terms of stroke timing, stroke occurred from the immediate postoperative period to 16 days later. The mean time to ischemic stroke was 2.94 days. A total of 56 (58.9%) patients developed strokes in the ICU within 2 days, and 39 (41.1%) had later strokes. There was a significant difference in stroke timing among the subgroups. Most LACI patients developed ischemic strokes later than patients in the other subgroups (*P*=0.045). A total of 36.4% of all LACI patients developed strokes within 48 h; the figures for TACI, PACI, and POCI patients were 76.5, 51.1, and 75%, respectively.

**Fig. 1:**
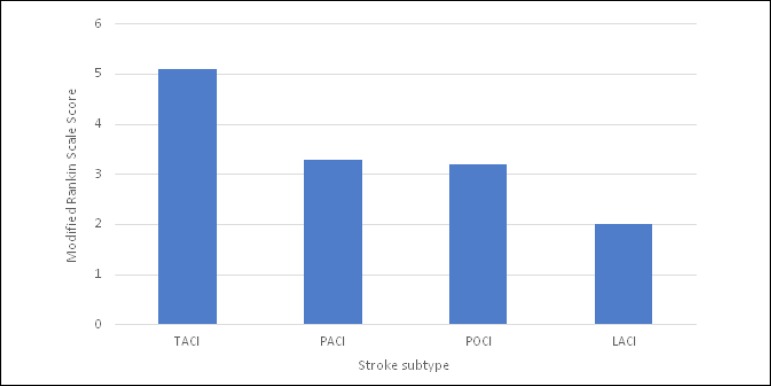
Modified Rankin Scale scores according to OCSP classification.

Twenty-eight (29.5%) patients died in hospital and six after discharge, to make a total of 34 deaths (35.8% of the cohort). Our results showed that both in-hospital and overall mortality (52.9% and 64.7%) were highest in the TACI group (*P*=0.125 and *P*=0.056, respectively). In between-subgroup comparisons, the overall mortality rate of the TACI group was significantly higher than that of the LACI group (64.7 *vs.* 27.3%, *P*=0.041). There was no significant difference in survival rates among the other groups (*P*>0.05). Also, the mean mRS score was highest in the TACI group (5.18±1.2); the Kruskal-Wallis test showed that this was significant (*P*=0.003) (Figure 1).

**Fig. 2:**
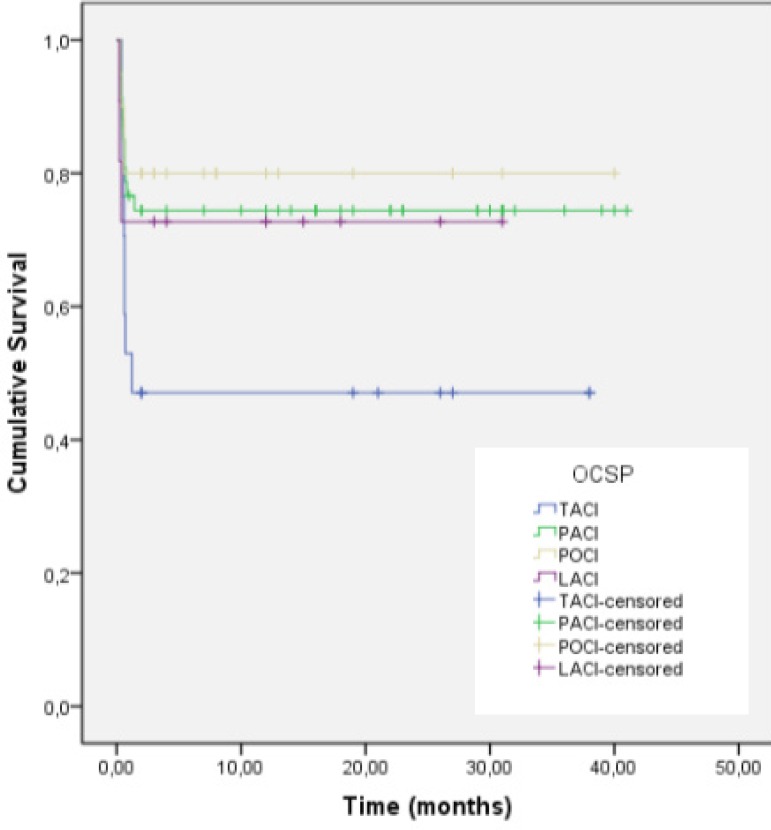
Kaplan-Meier survival analysis. LACI=lacunar infarction; OCSP=Oxfordshire Community Stroke Project classification; PACI=partial anterior circulation infarction; POCI=posterior circulation infarction; TACI=total anterior circulation infarction No significant difference in 5-year survival among the OCSP subgroups (P=0.285).

The mean cohort follow-up time was 30.1±1.6 months. The survival rates of the LACI (72.7%), PACI (70.2%), and POCI (70%) subgroups were similar, but that of the TACI subgroup was lower (35.3%). There was no significant difference in follow-up period between stroke subtypes (*P*=0.166) (Table 3). The log-rank test revealed no significant difference in 5-year survival among the OCSP subgroups (*P*=0.285; *P*>0.05). The Kaplan-Meier survival curve is shown in Figure 2.

**Table 3:** Stroke timing and major outcomes of the groups.

		OCSP Classification				
TACI (n=17)	PACI (n=47)	POCI (n=20)	LACI (n=11)	*P*
Stroke time	<48 hours	13 (76.5%)	24 (51.1%)	15 (75%)	4 (36.4%)	a0.045*
	>48 hours	4 (23.5%)	23 (48.9%)	5 (25%)	7 (63.6%)	
Modified Rankin Scale Score	Mean±SD	5.1±1.2	3.2±2.1	3.2±1.9	2.5±2.3	b0.003*
	Min-Max (Median)	2-6 (5)	0-6 (3)	1-6 (2.5)	0-6 (2)	
In-hospital mortality	Yes	9 (52.9%)	12 (25.5%)	4 (20%)	3 (27.3%)	a0.125
Overall mortality	Yes	11 (64.7%)	14 (29.8%)	6 (30%)	3 (27.3%)	a0.056
Follow-up	Months	23.1±3.6	30.2±2.1	33.7±3.9	24.8±2.6	

LACI=lacunar infarction; OCSP=Oxfordshire Community Stroke Project classification; PACI=partial anterior circulation infarction; POCI=posterior circulation infarction; TACI=total anterior circulation infarction

a Pearson chi-square test;

b Kruskall Wallis Test;

* *P*<0.05.

## DISCUSSION 

Our single-center retrospective study evaluated the stroke outcomes of a large cohort of patients undergoing elective on-pump CABG, by stroke subtype. Previous studies focused on risk factors associated with the timing of postoperative stroke after coronary surgery, but did not evaluate in-hospital or total mortality, the extent of follow-up, or the disability level by stroke subtype^[13,14]^. We evaluated 95 of 5380 consecutive CABG patients who experienced ischemic stroke by stroke-OCSP subtype.

Our study sample was relatively homogeneous. The mean age, gender distribution, and concomitant disease level were similar among patients differing in terms of OCSP stroke subtype. None of the pre-operative LVEF, hematocrit, or creatinine level differed among the stroke subtypes. Such homogeneity suggests that among-group morbidity and mortality comparisons may be valid.

We found that patients who developed different subtypes of ischemic stroke after CABG surgery experienced different outcomes. In overall and in-hospital mortality, and the extent of disability (measured using the mRS), were higher in the TACI subgroup than the other subgroups. In all subgroups, early stroke was more common and more serious then delayed stroke, and was particularly common in the TACI, PACI, and POCI subgroups. Delayed stroke was more frequent in the LACI subgroup; mortality was lower and the mRS score and survival were better in this subgroup. On follow-up, LACI-type stroke was associated with better 5-year and cumulative survival rates and a lower mRS score.

The overall prevalence of stroke was 1.76%, thus lower than the 2.7% reported by Hedberg et al.^[15]^. However, the in-hospital and total mortality of patients who developed ischemic stroke after CABG (n=95) were 29.5% and 35.8%, respectively. Santos et al.^[16]^ analyzed predictors of stroke in 4,626 patients after coronary surgery and noted that the incidence of stroke was 3% in those undergoing coronary surgery alone. However, mortality was 31.9% in the stroke group^[16]^. Our figures are similar to those in the literature (stroke incidence 1.76%; overall mortality 35.8%); such a high mortality rate is an enormous complication for surgeons and, of course, calamitous for patients. Also, the extent of dependence and disability after stroke is very challenging. Therefore, a clinical classification of postoperative stroke with prediction of outcomes is crucial.

In some previous studies, OCSP classification was used to rate patients with ischemic strokes (in the absence of coronary surgery)^[17,18]^. Paci et al.^[17]^ studied 8773 patients with ischemic stroke by OCSP classification. PACI was the most common subtype and the TACI subgroup experienced significantly higher mortality, similar to our study.

Patients developing early stroke had higher and earlier mortality than those with delayed strokes. In our study, the mean ischemic stroke time was 2.94 days postoperatively. The number of patients who developed early strokes was greater than the number who developed delayed strokes (58.9% *vs*. 41.1%). Also, our results showed that those who developed strokes within 48h had a higher mortality rate and mRS score. Hedberg et al.^[15]^ analyzed stroke developing after coronary surgery (early and delayed stroke) and concluded that stroke seriously increased the risk of short-term mortality, in line with our findings. However, they did not classify stroke by subtype. In our series, the TACI, PACI, and POCI subgroups had more early strokes (within 48 h): 76.5, 51.1, and 75%, respectively; the LACI subgroup had more delayed strokes (63.6%). Also, mortality was significantly lower in the LACI than the TACI subgroup (27.3% *vs.* 64.7%, *P*=0.041). Furthermore, the in-hospital mortality rate was higher in the TACI than the PACI, POCI, and LACI subgroups; 52.9% *vs. *25.5%, 20%, and 27.3%, respectively. Thus, when a stroke develops within 48h after coronary surgery, it is likely to be of the TACI form, associated with very high mortality and morbidity.

Our mean follow-up time was 30.1±1.6 months. The survival rates were similar in the LACI (72.7%), PACI (70.2%), and POCI (70%) subgroups, but lower in the TACI (35.3%) subgroup. There was no significant difference in the follow-up periods of patients with various stroke subtypes. We used the log-rank test to explore among-subgroup differences in 5-year survival, and found no such difference.

### Limitations

Stroke is a complicated, multifaceted clinical issue. We analyzed survival and disability, but we lacked information on quality of life because of retrospective study design. Also, mRS scores were calculated just prior to discharge; we have no long-term data regarding disability degree in the follow-up. However, our study is the largest clinical analysis by OCSP classification of stroke after CABG. Although in-hospital mortality was directly attributable to stroke-related events, total mortality was all-cause. We do not know whether later mortality was caused by stroke alone. Finally, we had no data on lesional size or volume, which may have influenced clinical outcomes.

## CONCLUSION

In conclusion, TACI subgroup patients had higher in-hospital and cumulative mortality rates and a higher mRS score. Strokes developing within 48h of coronary surgery were severe, especially in the TACI and POCI subgroups. The extent of dependency and disability were significantly higher in the TACI subgroup. We suggest that use of the OCSP classification and the mRS may render it possible to predict the outcomes of stroke after coronary bypass surgery.

**Table:** 

Authors' roles & responsibilities	
MA	Substantial contributions to the conception or design of the work; or the acquisition, analysis, or interpretation of data for the work; drafting the work or revising it critically for important intellectual content; final approval of the version to be published
CK	Substantial contributions to the conception or design of the work; or the acquisition, analysis, or interpretation of data for the work; final approval of the version to be published
MSB	Agreement to be accountable for all aspects of the work in ensuring that questions related to the accuracy or integrity of any part of the work are appropriately investigated and resolved; final approval of the version to be published
ZY	Substantial contributions to the conception or design of the work; or the acquisition, analysis, or interpretation of data for the work; final approval of the version to be published
AK	Agreement to be accountable for all aspects of the work in ensuring that questions related to the accuracy or integrity of any part of the work are appropriately investigated and resolved; final approval of the version to be published
AO	Agreement to be accountable for all aspects of the work in ensuring that questions related to the accuracy or integrity of any part of the work are appropriately investigated and resolved; final approval of the version to be published
MY	Final approval of the version to be published
TK	Final approval of the version to be published
BK	Drafting the work or revising it critically for important intellectual content; final approval of the version to be published
EA	Drafting the work or revising it critically for important intellectual content; final approval of the version to be published
